# Bacteria Peptidoglycan Promoted Breast Cancer Cell Invasiveness and Adhesiveness by Targeting Toll-Like Receptor 2 in the Cancer Cells

**DOI:** 10.1371/journal.pone.0010850

**Published:** 2010-05-26

**Authors:** Wenjie Xie, Yafang Huang, Wenling Xie, Aimin Guo, Wei Wu

**Affiliations:** 1 Biology Research Institute of the United Laboratories International Holdings Limited, Zhuhai, China; 2 School of Public Health and Family Medicine, Capital University of Medical Sciences, Beijing, China; 3 Xinjiang Modern Women Hospital, Urumqi, China; Health Canada, Canada

## Abstract

Chronic bacterial infection increased the risk of many solid malignancies and the underlying mechanism is usually ascribed to bacterial-caused inflammation. However, the direct interaction of infectious bacteria with cancer cells has been largely overlooked. We identified that highly metastatic breast cancer MDA-MB-231 cells expressed high level of Toll-like receptor 2 (TLR2) in contrast to poorly metastatic breast cancer cells and homogenous untransformed breast cells. TLR2 in MDA-MB-231 cells were actively triggered by peptidoglycan (PGN) from infectious bacterium *Staphylococcus aureus* (PGN-SA), resulting in the promoted invasiveness and adhesiveness of the cancer cells *in vitro*. PGN-SA induced phosphorylation of TAK1 and IκB in the TLR2-NF-κB pathway of the cancer cells and stimulated IL-6 and TGF-β secretion in MDA-MB-231 cells. All these effects were abrogated by TLR2 blockade. Further investigation showed that the NF-κB, STAT3 and Smad3 activities were augmented sequentially in MDA-MB-231 cells after PGN-SA stimulation. Phosphorylation of NF-κBp65 was initially increased and then followed by phosphorylation of STAT3 and Smad3 in the delayed 4 or 6 hours. NF-κB inhibition attenuated STAT3 and Smad3 activities whereas PGN-SA-stimulated cell culture supernatants reversed these inhibitory effects. Our study indicated that TLR2 activation by infectious bacterial PGN played an important role in breast cancer cell invasiveness and illustrated a new link between infectious bacteria and the cancer cells, suggesting the importance of antibiotic therapy to treat cancer with bacterial infection.

## Introduction

Metastasis seriously affects the survival of individuals with breast cancer[1]. Bacterial infection mastitis may aggravate this malignant metastasis [2], [3]. It is necessary to investigate the molecular mechanisms of breast cancer cell invasiveness and adhesiveness under the conditions of bacteria infection. Solid evidences have demonstrated that the underlying mechanism of chronic infection-associated tumorigenesis is definitively ascribed to pathogen-caused inflammation[4], [5]. For an example, in gastric cancer, *Helicobacter pylori* (*H.pylori*) plays a well-known risk factor of tumorigenesis duo to *H.pylori*-caused chronic inflammation[4], [6]. However, there were several reports showing that bacteria component lipopolysaccharide (LPS) could directly promote mouse colon tumor cell invasion and *listeria monocytogenes* could promote mouse hepatic tumor growth by interaction of pathogens directly with Toll-like receptors (TLRs) of tumor cells, despite mediation of pathogen-caused inflammatory microenviroment with infiltrating lymphocytes[7], [8]. Nevertheless, the interaction of bacteria with human cancer cells in invasiveness and adhesiveness remains unknown. We hypothesized that the effect of infectious bacteria directly on cancer cells could facilitate invasiveness and adhesiveness of the cancer cells.

Toll receptors were initially found in *Drosophila* as an important protein family which plays roles in dorso-ventral establishment of developing embryo and Pathogene-Associated Molecular Patterns (PAMPs) recognization for preventing fungal infection[9], [10]. Subsequently, mammalian homologues of Toll receptors, now termed Toll-Like Receptors (TLRs), were identified in immune cells as pattern-recognition receptors (PRRs) responsible for the detection of PAMPs in pathogene, and thus initiated innate immunity[9]. The TLR family in mammal consists of 11 members and each member recognizes different PAMPs of infectious microbial components[10]. For examples, TLR2 recognizes PAMPs in bacterial cellwall including bacterial lipopolysaccharide and peptidoglycan (PGN); TLR9 senses the PAMP of microbial plasmid, known as unmethylated DNA; TLR3 distinguishes the RNA of virus[11]. Recent studies identified several TLRs in non-immune system cells frequently involved in pathogenesis: TLR2 in mesenchymal stem cells playing a role in the control of cell differentiation[12]; TLR4 in adipocytes relating to insulin resistance[13]; TLR4 in hepatic stellate cells enhancing TGF-β signaling and hepatic fibrosis[14].

More recently, studies show several mouse and human tumor cells express certain TLR members and the TLR activation by bacteria or bacterial component seemed to promote tumor progression[8], [15], [16]. For examples, tumor cell TLR2 activation by *Listeria monocytogenes* promoted mouse hepatic tumor and human gastric carcinoma growth[8]; TLR9 activation by microbial unmethylated oligodeoxynucleotide (CpG-ODN) induced MDA-MB-231 cell invasion[16]. However, activation of TLR3 on human hepatic cancer cells directly induced their apoptosis[17]; Activation of rat prostate tumor cell TLR4 by *Ecoli* LPS inhibited tumor growth[18]. In present study, we found that breast cancer cells of different metastatic abilities expressed differential levels of TLR2 positively relating to cellular invasiveness and adhesiveness. The peptidoglycan of *Staphylococcus aureus* (PGN-SA) triggered breast cancer cell TLR2 with NF-κB, STAT3 and Smad3 activation contributing to the cancer cell invasiveness and adhesiveness. Our study illustrated a new TLR2-mediated pathway which disclosed the effects of bacterial component on breast cancer cell independent on infection-associated inflammation.

## Methods

### Reagents and antibodies

Matrigel and fibronectin were purchased form Becton Dickincon Biosciences (San Jose, CA, USA). PGN-SA (one of TLR2 ligands, peptidoglycan from *Staphylococcus aureus*), PGN-EB (one of TLR2 ligands, peptidoglycan from *Ecoli 0111:B4 strain*), LPS-EB (one of TLR4 ligands, lipopolysaccharide from *Ecoli 0111:B4*) and neutralizing monoclonal antibody to human TLR2 were purchased from Invivogen (San Diego, CA, USA). FITC-conjugated primary anti-TLR2 monoclonal antibody and its isotype antibody FITC-IgG2a were all purchased from eBiosciense Inc (San Diego, CA, USA). SN50, a cell-permeable NF-κB inhibition peptide, was purchased from Calbiochem (EMD Biosciences, Inc. San Diego, CA, USA). Monoclonal anti-pNF-κB p65-S536 antibody and anti-pSmad3-S423/425 antibody were purchased form Cell Signaling Technology, Inc. (Danvers, MA, USA). PE-conjugated anti-pSTAT3-Y705 antibody was obtained from Becton Dickincon Biosciences (San Jose, CA, USA). FITC-conjugated secondary antibody of goat anti-rabbit IgG (H+L) was purchased from BioLegend (San Diego, CA, USA). Rabbit IgG, mouse IgG and PE-conjugated primary mouse antibody IgG2a were obtained from Santa Cruz Biotechnology, Inc. (Santa Cruz, CA, USA).

### Cell lines and cell culture

Human breast adenocarcinoma cell line MCF-7 (ATCC, NO HTB-22), MDA-MB-468 (ATCC, NO HTB-22), MDA-MB-231(ATCC, NO HTB-26) and nontransformed breast cell MCF-10A(ATCC, NO HTB-26) were purchased from American Type Culture Collection (ATCC; Rockville, MD, USA). These cell lines were All cultured in Dulbecco's modified Eagle's high glucose Medium supplemented with 10% FBS (Hyclone). The medium was replaced every 2 days and once 80% confluent layer was formed, the cells were removed using trypsin (0.05% EDTA, 0.25% trypsin; Sigma) and reseeded.

### Evaluation of TLR2 expression

Flow cytometry (MOFLO, DAKO, Inc. CO, USA) was used to quantify TLR2 expression. The two cell lines were separately stained with FITC-conjugated TLR2 antibody or isotype IgG according to the manufacturer's instructions. Cells were analyzed with a MoFlo flow cytometer and the data were analyzed by Summit Software v4.3 (DAKO, Inc. CO, USA).

### Real time-PCR to analyze the expression of TLR2 mRNA

Total RNA was extracted by a single-step method with TRIzol reagent (Invitrogen Corp., Carlsbad, CA, USA). For measuring RNA concentration and protein contamination, absorbance of RNA solution was measured at OD 260 and 280 nm. The reverse transcriptase reaction was carried out using avian myeloblastosis virus (AMV) reverse transcriptase (Promega, Madison, WI, USA). PCRs were performed according to the real time PCR machine manufacturer's instructions (ABI 7300), which allow real time quantitative detection of the PCR product by measuring the increase in SYBR green fluorescence caused by binding of SYBR green to double-stranded DNA. The SYBR kit for PCRs was purchased from PerkinElmer Life Sciences. TLR2 mRNA yield differences were calculated using GAPDH as endogenous control and the comparative threshold cycle (Ct value)[19]. Primers for human TLR2 and β-actin were designed were designed according to I. Kokkinopoulos[20].

### Transwell invasion assay

The bottoms of the inner transwells (8.0 µm pore size, 6.5 mm diameter; Corning Costar, Inc. CA, USA) were coated with paraffin to avoid meniscus formation. Then, the bottoms of the transwells were coated with a thin layer of matrigel (2.5 mg/ml). The two cell lines in DMEM containing 0.2% BSA were seeded on top of this matrigel at a quantity of 50,000 cells per chamber. The cells were cultured in serum-free medium in the inner compartment inside the transwell. To the outer compartment outside the transwell, we added 600 µl DMEM with 10% FBS. After 24 h culture, the cells were fixed with 2% paraformaldehyde in 1×PBS and stained with 0.05% toluidine blue in 2% Na_2_CO_3_/water solution. After wiping off the inside of the chambers using cotton-tipped applicators, invasive cells remained on the bottom of the membranes and were counted under the microscope (magnification, ×200)[21].

### *In vitro* adhesion to fibronectin assay

Twenty-four-well culture dishes were pre-coated with 80 µl of Fibronectin (2.5 mg/ml) adhesion buffer (0.25% BSA in HBSS) for 30 min at 37°C. 1×10^5^ cancer cells were added to each well. After 30 min at 37°C in a CO2 incubator, nonadherent cells were removed by gentle wash with HBSS. Then, the cells were fixed with 2% paraformaldehyde in 1×PBS and stained with 0.05% toluidine blue in 2% Na_2_CO_3_ water solution. The adherent cells of the bottom were counted under the microscope (magnification, ×100).

### ELISA

1×10^5^ cancer cells were cultured in 24-well plates for 24 h and the supernatants were assayed for IL-6, TGF-β, VEGF and MMP9 using an ELISA kit (R&D Systems, Minneapolis, MN, USA) according to the manufacturer's instructions.

### Western-blotting

For pTAK1 or pIκBα detection, the cells were resuspended in lysis buffer (10 mM Tris–HCl, pH 8.0, 137 mM NaCl, 10% glycerol, 1% Triton X-100, 1 mM Na_3_VO_4_, 1 mM phenylmethylsulfonyl fluoride, 10 µg/ml leupeptin, 1 µg/ml aprotinin). For TLR2 detection, the cells were lysed in RIPA buffer (50 mM HEPES, 150 mM NaCl, 1% deoxycholate, 1% NP-40, 0.5% SDS and protease inhibitor). The cell lysates were centrifuged for 20 min at 16,000 g, and then equal amounts of protein (30 µg) were separated by 10% SDS-PAGE, and transferred onto PVDF membranes (Millipore, Bedford MA, USA). After blocking in non-fat dried milk, membranes were probed with primary antibody in a solution of PBS containing 0.1% Tween20. After three 10-min wash in PBS/0.1% Tween20, blots were incubated with horseradish-peroxidase-conjugated secondary antibody. Immunoreactive bands were detected using the enhanced chemiluminescence system (Amersham, Piscataway, NJ, USA).

### Intracellular phospho-protein staining techniques for flow cytometer analysis

Formaldehyde in 0.5–1 ml PBS was added to each cell sample, to a final concentration of 2%, and cells were fixed for 10 min at 37°C. To make cells permeable, they were slowly added to ice-cold 100% methanol to a final concentration of 90%. Then, cells were incubated for 30 min on ice. Two milliliters of 2% BSA blocking buffer was added to each tube, and tubes were rinsed by centrifugation at 350 g. The permeabilized cells were resuspended in 100 µl blocking buffer and primary antibody was added to each tube, which was incubated on ice for 45 min. After two wash in blocking buffer to delete antibodies with non-specific attachment, the cells were resuspended in FITC-conjugated secondary antibody. After 30 min, cells were rinsed again and resuspended in 0.5 ml PBS for analysis by MoFlo flow cytometer (DAKO, Inc. CO, USA). The strength of fluorescence was measured by the median fluorescence intensity (MFI). Fold change was calculated by dividing the MFI of the treated sample (MFI treated) from that of the control sample (MFI untreated), i.e., fold change percent % = (MFI treated/MFI untreated) ×100[22].

### Statistical analysis

The values were expressed as mean ± SD. Statistical comparison was made using Student's *t* test, and the level of significance was established at *p*<0.05 and *p*<0.01.

## Results

### TLR2 was differentially expressed in breast cancer cell lines

**Figure 1:**
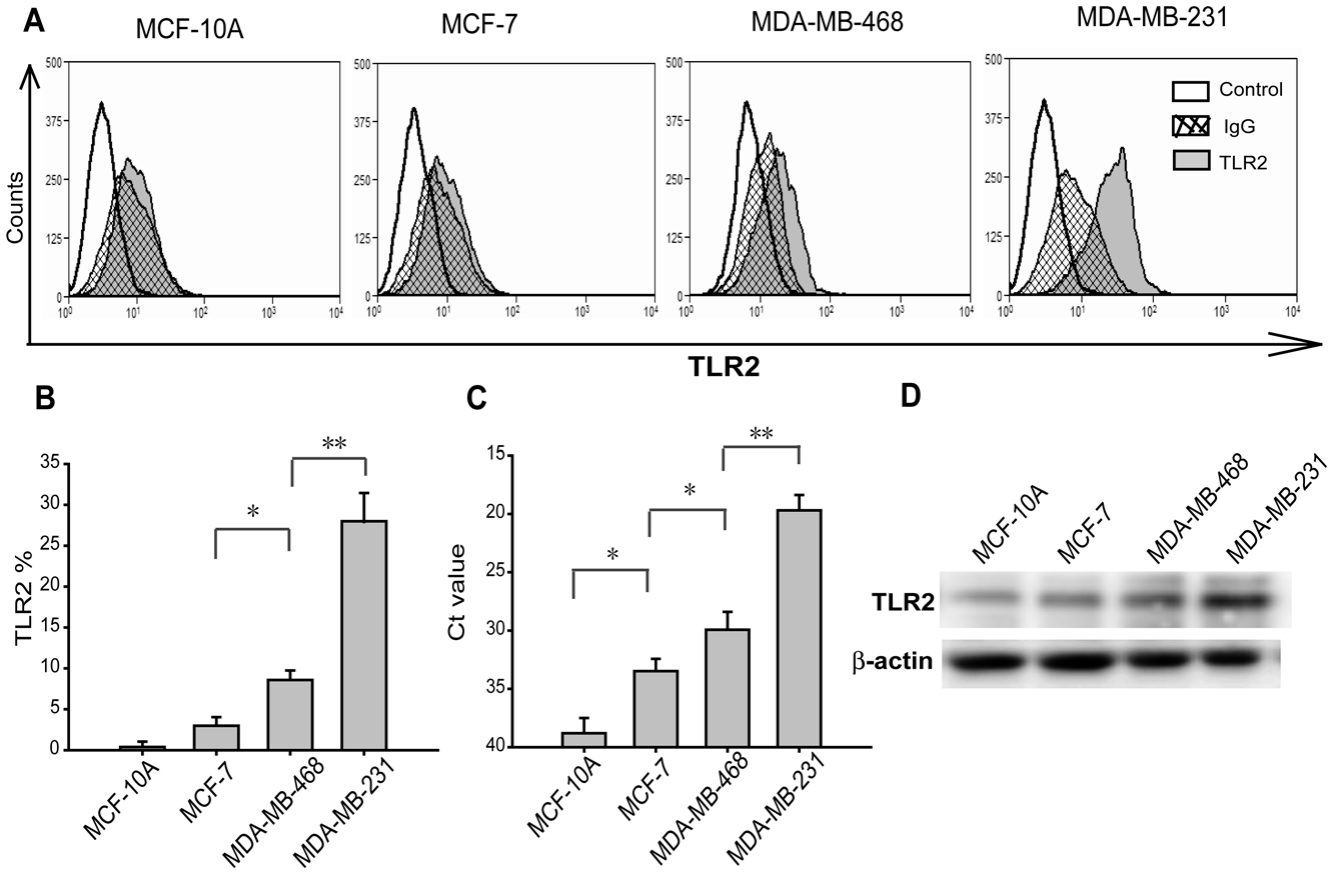
TLR2 was differentially expressed in breast cancer cells and untransformed breast cells. (A and B) Surface expression of TLR2 in four cell lines was analyzed by direct immunofluorescence using flow cytometer and IgG was used as control antibodies. The differences of TLR2 expression levels in breast cancer MCF-7, MDA-MB-468 and MDA-MB-231 cells were significant and TLR2 in untransformed MCF-10A cells was nearly undetectable. (C) TLR2 mRNA levels in the cell lines were measured by real-time PCR. RNA yield differences were calculated using GAPDH as an endogenous control and the comparative threshold cycle (Ct value). MDA-MB-231 cells showed the lowest Ct value among these cell lines. (D) TLR2 protein levels were analyzed by Western-blotting. Values were expressed as mean ± SD (*n* = 6 *p<0.05; **p<0.01).

Previous report pointed that mouse hepatoma H22 cells expressed TLR2 receptor involved in tumor growth by *listeria monocytogenes* stimulation[8]. We sought to determine the effect of TLR2 stimulation with infectious bacteria on breast tumor invasiveness and adhesiveness. Therefore, we initially detected TLR2 expression levels of selected breast cancer cell lines. By flow cytometer detection, we found TLR2 was differentially expressed in MCF-10A, MCF-7, MDA-MB-468 and MDA-MB-231. Qualitative PCR analysis indicated the similar result. TLR2 protein level in MDA-MB-231 cells was much higher than the other cell lines (**Fig. 1A and B**). Real-time PCR analysis showed the number of threshold cycles (Ct) of MDA-MB-231 was the least among those of other three cell lines (**Fig. 1C**). These results were confirmed by western blot analysis (**Fig. 1D**). Since each of these cell lines is distinguished in invasive and metastatic capacities, differential expression of TLR2 in those cell lines might be related to their metastatic capacity.

### PGN-SA promoted breast cancer cell invasiveness and adhesiveness via TLR2

**Figure 2:**
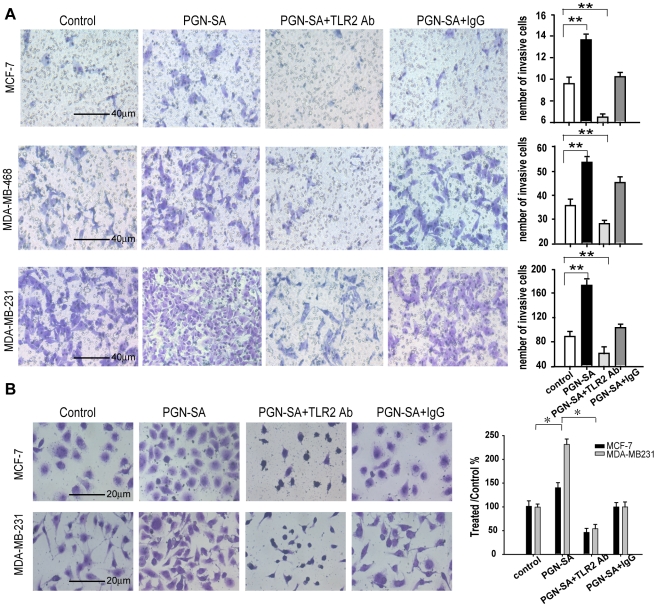
PGN-SA promoted invasiveness and adhesiveness via TLR2 in MDA-MB-231 cells. (A) The bottoms of the top cells in Transwell setting were all coated with a thin layer of matrigel and only the cells with invasive capacity could migrate through the matrigel layer and 8.0 µm pore. IgG (10 µg/ml) treatment was a antibodies control. TLR2 activation by PGN-SA (1 µg/ml) increased the invasive cell number of breast cancer cell lines whereas TLR2 inhibition by anti-TLR2 neutralizing antibody (10 µg/ml) attenuated the invasive cells. (B) The culture dishes were pre-coated with 80 µl of fibronectin (2.5 mg/ml). The adhesive cancer cells stained with 0.05% toluidine blue solution. PGN-SA (1 µg/ml) enhanced MDA-MB-231 cell adhesion to fibronectin layer whereas anti-TLR2 neutralizing antibody (10 µg/ml) abrogated PGN-stimulated adhesive ability. Values were expressed as mean ± SD and *in vitro* experiments, one representative of six independent is shown (n = 6 *p<0.05; **p<0.01).

Metastatic process involves several critical steps such as invasion and adhesion[23]. To investigate the effects of TLR2 on the metastatic process of the breast cancer cell lines, the Transwell experiment for invasion assay was set up and only highly invasive cells can invade through the matrigel layer and migrate through the 8.0 µm holes[21]. Among the three breast cancer cell lines, MDA-MB-231 cells showed highest capacity in invasion (**Fig. 2A**). Utilizing TLR2 agonist PGN-SA (1 µg/ml) to activate TLR2 and anti-TLR2 neutralizing antibody to block TLR2, we found that in MDA-MB-231 cells, PGN-SA stimulation promoted more than 2-fold invasive capacity than the unthreatment (**Fig. 2A**). On the contrary, anti-TLR2 neutralizing antibody notably attenuated the number of invasive breast cancer cells (**Fig. 2A**). Adhesion to extracellular matrix (ECM) is an important capacity for cancer cell to anchor on the matrix[23]. We deploy fibronectin, an important component of ECM, to investigate the effect of TLR2 activation on MDA-MB-231 cell adhesiveness to this kind matrix. PGN-SA significantly enhanced the adhesion of MDA-MB-231 cells whereas there is no such a similar effect in MCF-7 cells by PGN-SA stimulation (**Fig. 2B**).

### TLR2 activation by PGN-SA-triggered NF-κB activity

**Figure 3:**
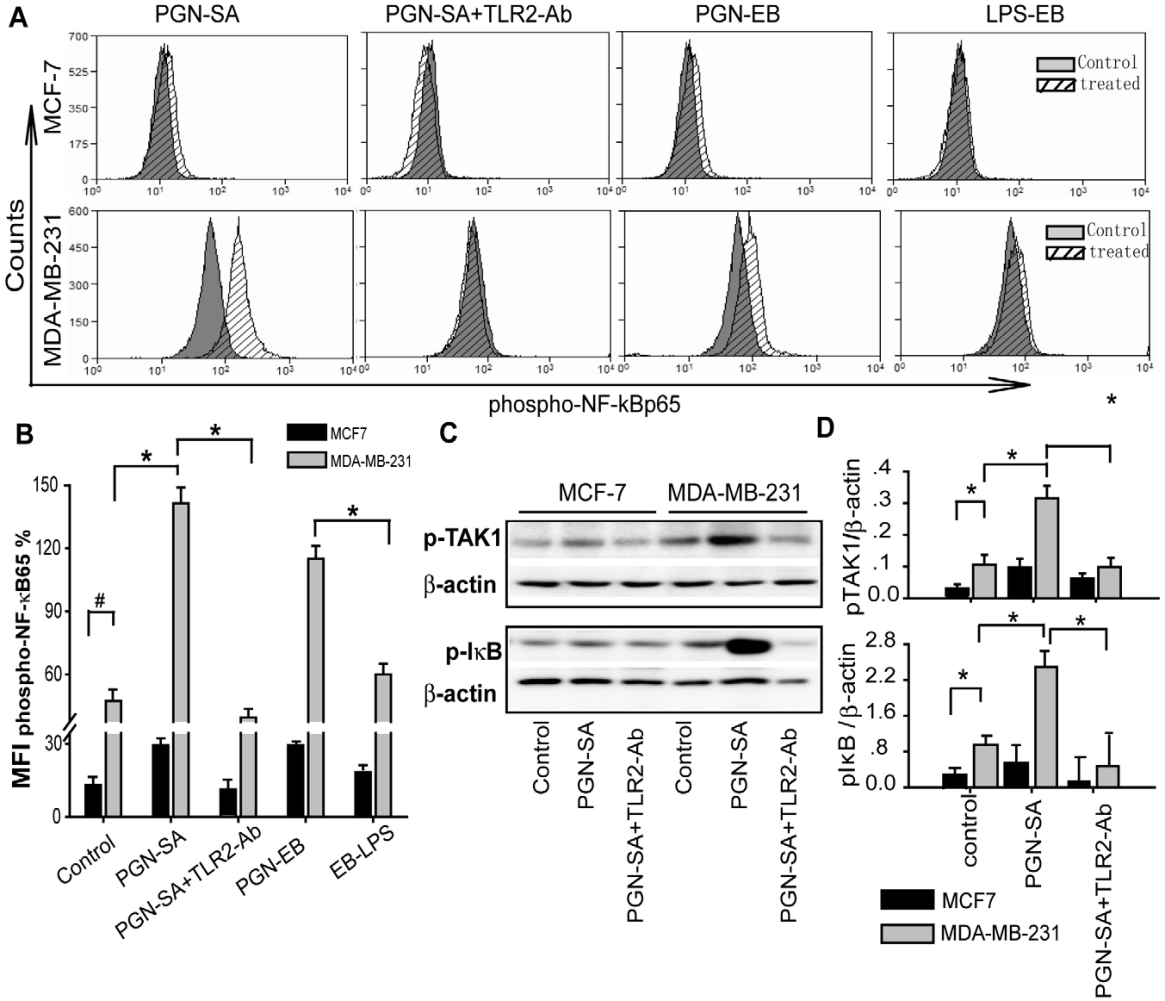
Bacterial component PGN-SA enhanced TLR2-NF-κB signaling in breast cancer cells. (A and B) Specific expression of the phospho-NF-κBp65 was detected in cancer cells using flow cytometry assay, for which equal volume of PBS treatment served as a control. PGN-SA (1 µg/ml) and PGN-EB (1 µg/ml) promoted augment of NF-κB activation in MDA-MB-231 cells nearly 4-fold more than MCF-7 cells. LPS-EB (1 µg/ml), a TLR4 agonist, had no such an effect. TLR2 blockade by its neutralizing antibody (10 µg/ml) abrogated PGN-SA-stimulated NF-κB activities in both MDA-MB-231 and MCF-7 cells. (C and D) PGN-SA increased amounts of phospho-TAK1 and phospho-IkBα in MDA-MB-231 cells in contrast to MCF-7 cells. Values were expressed as mean ± SD and one representative of six independent experiments was shown (n = 6 *p<0.05; **p<0.01; #*p*<0.05).

NF-κB activition has been found in breast cancer cell lines and plays a pivotal role in metastatic progress of breast cancer cells[24], [25]. In immune cells, TLR2 stimulation enhances NF-κB activation[26]. We therefore investigated whether TLR2 activation in human breast cancer cells leads to NF-κB activity. By PGN-SA stimulation, MDA-MB-231 cells up-regulated NF-κB activity 4-fold stronger whereas TLR2 blockade by anti-TLR2 neutralizing antibody significantly decreased NF-κB activity (**Fig. 3A and B**). PGN of *Ecoli 0111:B4* (PGN-EB), another TLR2 agonist, also increased NF-κB activity in MDA-MB-231 cells, but such a response of NF-κB activity was not caused by LPS-EB (a TLR4 agonist) (**Fig. 3A and B**). Nevertheless, PGN-SA and PGN-EB could not trigger NF-κB activity in poorly metastatic and TLR2-low expressed MCF-7 cells (**Fig. 3A and B**). These results indicated that highly metastatic breast cancer cell MDA-MB-231 cells interacted with the bacterial PAMPs by TLR2 but not TLR4, and thus leaded to NF-κB to activation. The poorly metastatic MCF-7 cells could not result in the similar effect. To confirm the signal pathways of TLR2-NF-κB, we further investigated pTAK1 and pIkBα, the two critical kinases in TLR2-NF-κB signaling[26]. The result showed that after PGN-SA stimulation, phosphorylation level of TAK1 and IkBα in MDA-MB-231 cells were significantly increased but the parallel test for MCF-7 cells did not appear significant increase (**Fig. 3C** **.D**).

### TLR2 activation promoted NF-κB activation and distinguished IL-6 and TGF-β secretion in invasive MDA-MB-231 cells

**Figure 4:**
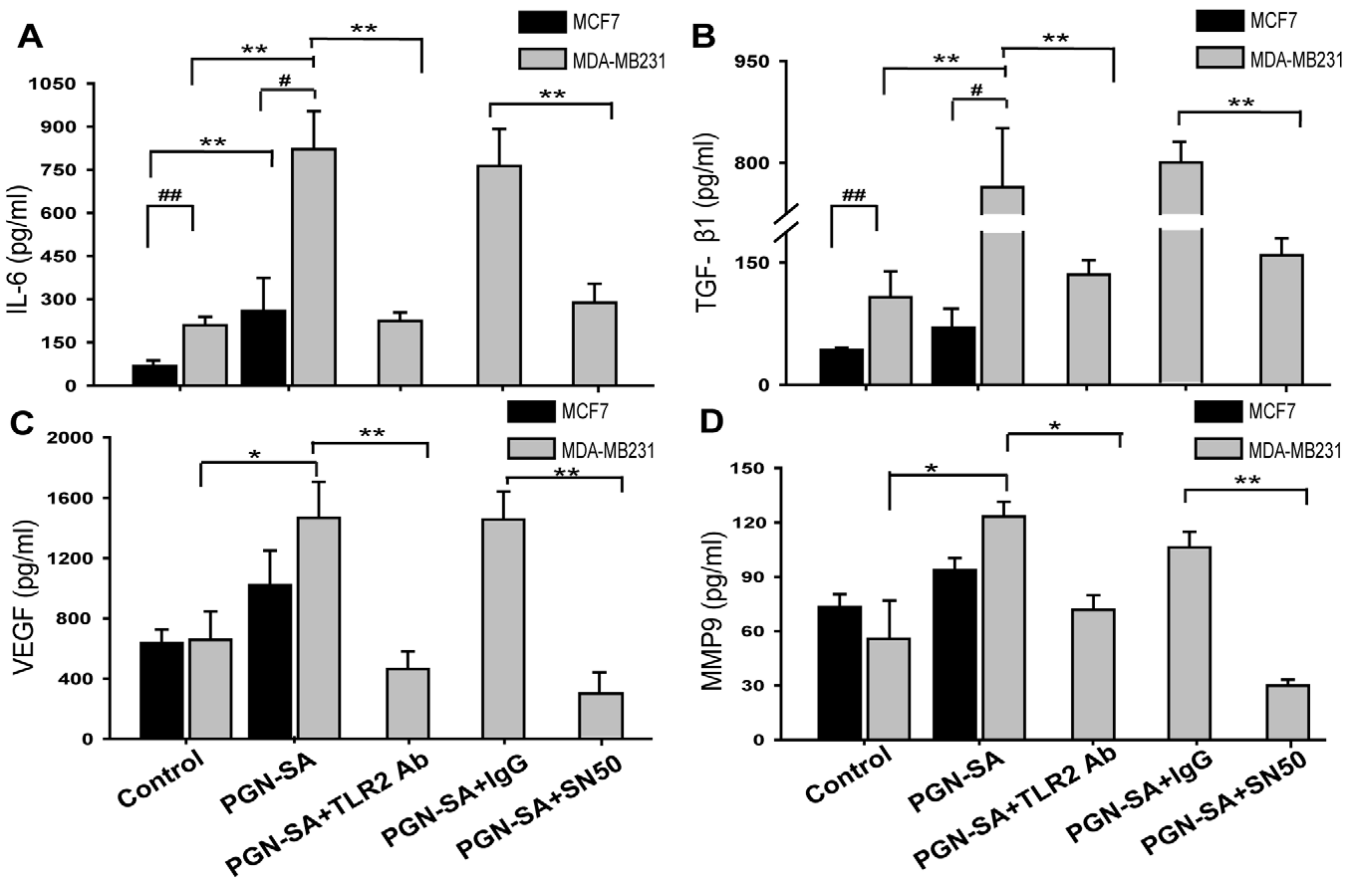
TLR2 activation by PGN-SA increased IL-6, TGF-β, VEGF and MMP9 secretion. (A and B) Cancer cells were cultured for 24 hours and the supernatants were analyzed by ELISA assay. MDA-MB-231 cells with PGN-SA (1 µg/ml) stimulation secreted IL-6 and TGF-β 4-folds more than untreated cells whereas TLR2 blockade by its neutralizing antibody (10 µg/ml) reduced IL-6 and TGF-β secretion obviously. (C and D) PGN-SA stimulation significantly up-regulated VEGF and MMP9 secretion in both MDA-MB-231 and MCF-7 cells. Blockade of TLR2 attenuated amounts of VEGF and MMP9 in MDA-MB-231. (A, B, C and D) NF-κB cell-permeable inhibitor peptide SN50 (10 µg/ml) abrogated these cytokine increment by PGN-SA stimulation. IgG (10 µg/ml) served as antibody control. Values were expressed as mean ± SD (n = 6 *P<0.05; **P<0.01; *^#^P*<0.05; *^##^P*<0.01).

The results above indicated that TLR2 activation by bacterial PAMPs of PGN promoted breast cancer cell invasiveness and adhesiveness as well as TLR2-NF-κB signalling activation. Cancer cell NF-κB activation could lead to abnormal cytokine transcription and thus regulate the cancer cell biology in metastatic process[27], [28], [29]. For example, NF-κB-mediated IL-6 promotes cancer cell proliferation and invasion[29]. We examined the cytokines secreted by MDA-MB-231 cells in culture supernatants involving IL-6, TGF-β, VEGF and MMP9 which are required for cancer cellular invasion and metastasis[30], [31], [32], [33]. Among the cytokines we investigated, IL-6 and TGF-β increased up to 5-folds and 7-folds respectively after TLR2 stimulation with PGN-SA (**Fig. 4A and B**). However, TLR2 blockade reduced IL-6 and TGF-β (**Fig. 4A and B**). Although the PGN-SA-induced increment of VEGF and MMP9 also appeared, the secretion quantitative of these cytokines was not different between MCF-7 and MDA-MB-231 cells (**Fig. 4C and D**). Differentially, IL-6 and TGF-β secreted by MDA-MB-231 cells under the condition of PGN-SA stimulation were up to 3-fold higher than MCF-7 (**Fig. 4A and B**).

### TLR2 activation triggers STAT3 and Smad3 activation

**Figure 5:**
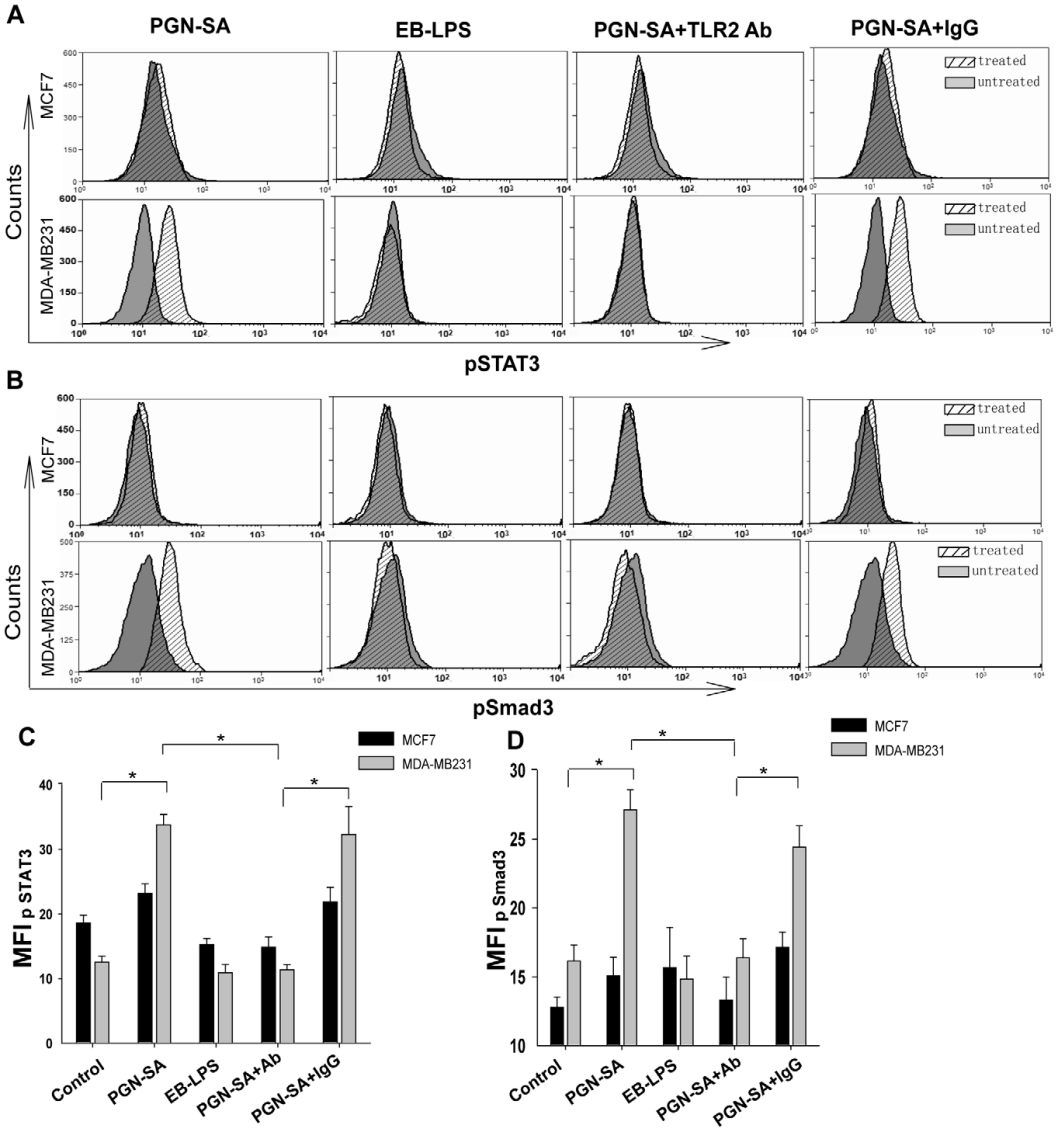
PGN-SA stimulation induced STAT3 and Smad3 activation via TLR2. (A, B, C and D) Phospho-STAT3 and Phospho-Smad3 were detected in cancer cells using flow cytometry assay and equal volume of PBS treatment served as a control. STAT3 and Smad3 activation were all enhanced by PGN-SA stimulation whereas TLR2 blockade hampered STAT3 and Smad3 activation. (C and D) On contrast to TLR2-low cell line MCF-7, MDA-MB-231 cells showed more sensitive to PGN-SA stimulation. Values were expressed as mean ± SD (n = 6 *P<0.05; **P<0.01).

From the results above, the secretion of IL-6 and TGF-β in MDA-MB-231 cells were in higher level than in MCF-7 cells. To determine the consequence of the IL-6 and TGF-β increment, we investigated the cytokine-coupled transcriptional factor signals of STAT3 and Smad3 which were identified as important factors responsible for malign tumor progress[34], [35]. Phosphorylation analysis by flow cytometer revealed that in MDA-MB-231 cells, STAT3 and Smad3 activities were all enhanced by PGN-SA stimulation whereas TLR2 blockade significantly attenuated STAT3 and Smad3 activities (**Fig. 5A–D**). In stead, TLR2-low MCF-7 cells did not show significantly variance in Smad3 and STAT3 activation. In addition, blockade of TLR2 abrogated PGN-SA-induced STAT3 and Smad3 activation.

### TLR2 activation sequentially stimulates NF-κB, STAT3 and Smad3 activation

**Figure 6:**
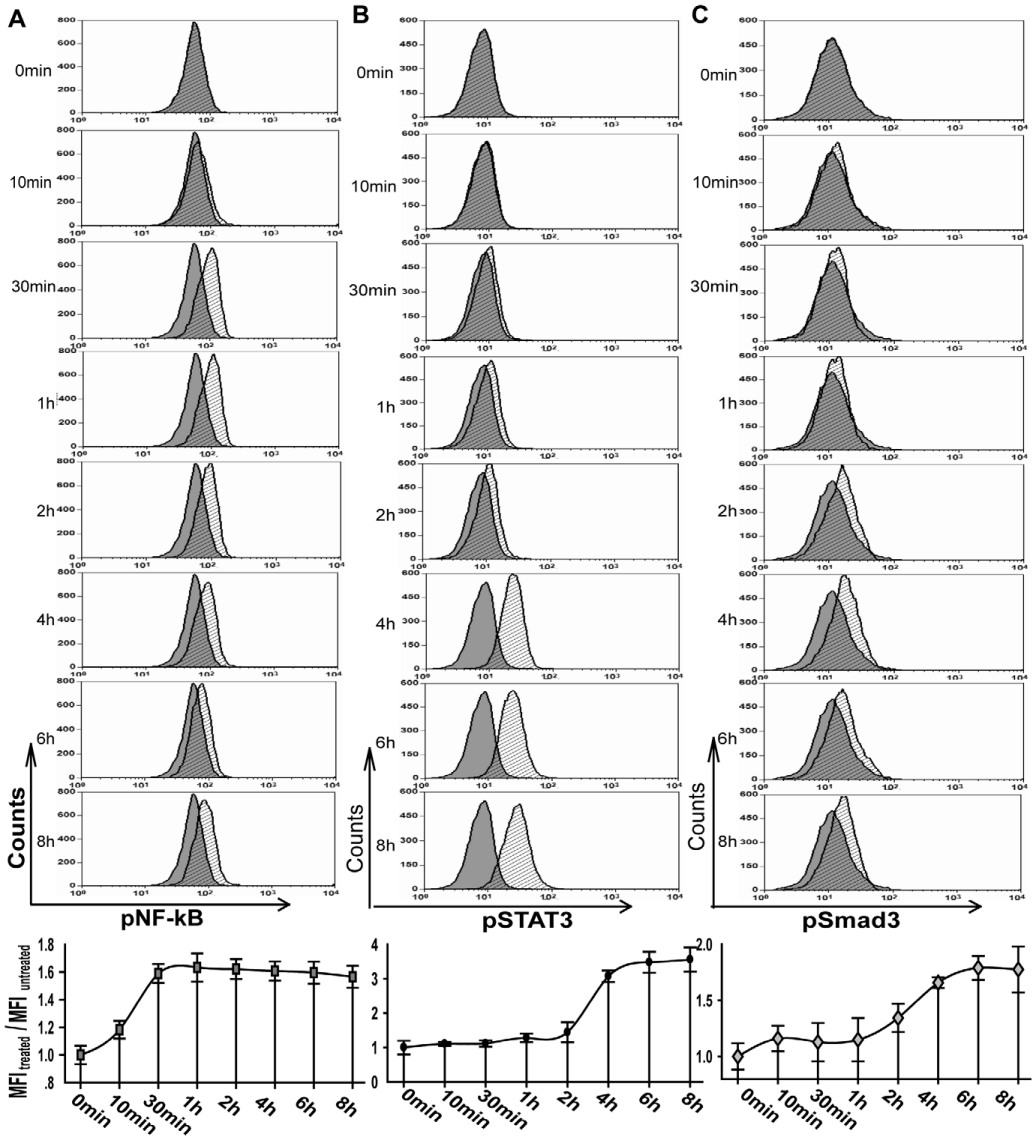
PGN-SA induced NF-κB, STAT3 and Smad3 activation sequentially in MDA-MB-231 cells. (A) The cancer cells were incubated with PGN-SA on the indicated time points of 0 min, 10 min, 30 min, 1 h, 2 h, 4 h, 6 h, 8 h. Anti-phospho-NF-κBp65, anti-Phospho-STAT3 and anti-Phospho-Smad3 were respectively used to mark the special target in permeabilized MDA-MB-231 cells. On 30 min, NF-κB was firstly stimulated up to maximum level. (B and C) After additional 4 h or 6 h, activations of STAT3 and Smad3 were respectively induced. Values were expressed as mean ± SD (n = 3 *P<0.05; **P<0.01).

Recent reports show that NF-κB activation is essential for breast cancer metastasis due to their contribution to epithelial-mesenchymal transition (EMT) and anti-apoptosis effect[24]. In addition, STAT3 and Smad3 activations were also necessary to the metastatic steps such as invasion and proliferation[34], [35]. Our study above showed that PAMPs of bacterial peptidoglycan triggered NF-κB, STAT3 and Smad3 activities via TLR2 pathway. To determine the mechanisms of these transcriptional factors responding to bacterial PAMPs, we analysed phosphorylation levels of these transcriptional factors on arrange of time points. On different time points, three transcriptional factors showed sequential activation after receiving bacterial PAMPs stimulation (**Fig. 6A–D**). On 30 min, phosphorylation of NF-κB was firstly triggered up to maximum level by PGN-SA (**Fig. 6A**). Then, after additional 4 h and 6 h, phosphorylation level of STAT3 and Smad3 were sequentially induced to near peak value (**Fig. 6B and C**). These results indicated that TLR2-associated NF-κB, STAT3 and Smad3 activities depended on a temporal manner mechanism, suggesting that NF-κB activity was responsible for sequential STAT3 and Smad3 activation.

### NF-κB activation is essential for STAT3 and Smad3 activation

**Figure 7:**
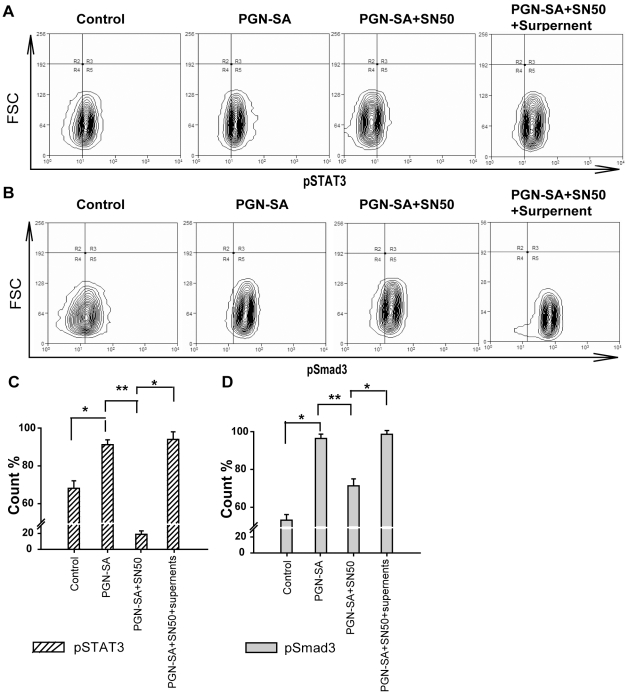
NF-κB activation is essential for STAT3 and Smad3 activation in breast cancer cells. (A, B and C) PGN-SA challenge increased the levels of STAT3 and Smad3 activation whereas NF-κB inhibitor SN50 (10 µg/ml) abrogated these effects of PGN-SA stimulation. After the cells incubated with PGN-SA for 6 h, PGN-SA-stimulated cell culture supernatant replaced the supernatant of PGN-SA+SN50 group and in this group (PGN-SA+SN50+Surpnatant), the STAT3 and Smad3 activities were increased to the levels of PGN-SA treatment once more. Values were expressed as mean ± SD (n = 6 *P<0.05; **P<0.01).

To determine whether STAT3 and Smad3 sequential activities required NF-κB activation, we deployed a NF-κB inhibitor, cell-permeable peptide SN50, to observe the consequence of NF-κB inhibition. The results showed that SN50 significantly decreased PGN-SA-induced STAT3 and Smad3 activities (**Fig. 7A–C**). These results demonstrated that inhibition of NF-κB could abrogate PGN-SA-induced STAT3 and Smad3 activities and the NF-κB activity was required for augment of STAT3 and Smad3 activities by PGN-SA stimulation. Since PGN-SA stimulation enhanced secretion of IL-6 and TGF-β in culture supernatant, we used PGN-SA-stimulated cell culture supernatant to replace the supernatant of PGN-SA+SN50 treated cells. Notably, we found that PGN-SA-stimulated supernatant treatment reversed the STAT3 and Smad3 activity regression by SN50 inhibition (**Fig. 7A–C**). Combined with ELISA results above, these results illustrated that PGN-SA-stimulated NF-κB in MDA-MB-231 cells resulted in releasing IL-6 and TGF-β and thus promoted STAT3 and Smad3 activity whereas if NF-κB was inhibited, the IL-6 and TGF-β as well as their coupled transcript factor STAT3 and Smad3 activities were also deduced.

## Discussion

In this study, we identified that TLR2 was differentially expressed in breast epithelial cancer cell lines and homogenous untransformed breast cell lines. TLR2 expression levels in these cell lines were consistent with their differential metastastic capacities. Toll-like receptors(TLRs) are sensitive to pathogene-associated molecular patterns (PAMPs) and damage-associated molecular patterns (DAMPs) released by pathogen and injured tissue[36], [37], [38]. Recent accumulated studies show TLRs in the cells of different tissue plays an important role in pathogenesis of many diseases[14], [37]. For example, TLR4 signaling in hepatic stellate cells enhanced TGF-β secretion and hepatic fibrosis[14]. Moreover, tumor cells also expressed certain member of TLR family leading to response to bacterial component. For examples, Gram-positive bacterium *Listeria monocytogene* accelerated mouse hepatocarcinoma cell H22 growth via TLR2 signaling[8]; TLR9 agonist CpG ODN promotes breast cancer cell MDA-MB-231 to activation of invasion via increased MMP9 secretion[16]. Our study shows that compared with nearly undetectable TLR2 level in untransformed breast cells, classic highly metastatic breast cancer MDA-MB-231 cells express high level of TLR2. Cancer cell TLR2 activation by peptidoglycan of pathogenic bacterium *Staphylococcus aureus* resulted in enhancement of invasiveness and adhesiveness. However, TLR2 blockade abrogated such an effect. As a pathogenic bacterium frequently contains both TLR2 agonist[11], our results indicated that breast cancer invasiveness and adhesiveness would be enhanced after the stimulation by peptidoglycan in bacteria and the highly metastatic breast cancer cells are more sensitive to pathogenic bacterial peptidoglycan than poorly metastatic breast cancer cells.

Bacterial infection or bacterial contamination following tumor surgery has been shown to be a risk factor of tumor metastatic recurrence, the underlying mechanisms is ascribed to infection-induced inflammation[3], [39], [40], [41]. Nevertheless, direct interaction between bacteria and tumor cells has been largely overlooked. In a murine model of metastasis, animals subjected to laparotomy or air laparoscopy with high levels of Endotoxin/LPS contamination had increased lung metastatic burden[7]. In the present study, we provided new insight into the relationship between bacteria infection and metastasis. Since the bacterial component peptidoglycan directly stimulated breast cancer cells invasiveness and adhesiveness via cancer cell TLR2 activation, our results suggest breast cancer patient might be necessarily noticed mastectomy or mastitis-caused bacterial infection, especially induced by Gram-positive bacteria which usually enriched much more peptidoglycan, and indicate the significance for bacterial infection-associated cancer to receive antibiotic therapy. Since common bacterial vaccine may contain TLR2 agonist, attention should also be paid to the effects of bacterial vaccines for immunotherapy on cancer cells. These require a design and genetically modified strategy to develop bacterial vaccine with reduced TLR2-activating potential.

We further report that PGN-SA elicited an augment of constitutive NF-κB activity via activation of TLR2 in breast cancer cells whereas TLR2 blockade or NF-κB activity inhibition decreased these cytokine secretions. Solid evidence has verified NF-κB as an essential factor in breast cancer metastasis[24], [29]. Recent reports emphasized that strong constitutive NF-κB activity in the highly invasive breast cancer MDA-MB-231 cell line compared with several breast cancer cell lines that are poorly invasive and metastatic[25]. However, the factors which induce NF-κB different activities in invasive or non-invasive breast cancer cells have not yet been elucidated. Our results indicated that PGN-SA powerfully mediated NF-κB higher activation in invasive MDA-MB-231 cells than in non-invasive MCF-7 cells due to differential expression of TLR2. In addition, PGN-SA triggered increase of pTAK1 and pIκBα which are two key kinases in the TLR2-NF-κB signal pathway that exists in immunocells and epithelial cells[26], and these results validated a TLR2-NF-κB signaling in invasive MDA-MB-231 cells. Accumulated evidences showed that the consequence of NF-κB signaling associated with cytokine secretion and cytokines secreted by cancer cells exerted an important effect on control of cancer cell biology such as proliferation, EMT and invasion[42]. For examples, IL-6 promotes cancer cell proliferation and recent studies showed that IL-6 also contributed to cancer cell malignant properties and effusion respectively in lung and breast cancer[43], [44]; TGF-β induces cancer cell invasion and EMT in breast cancer[27], [45]; VEGF and MMP9 are also regarded as indispensable cytokines for breast cancer cell invasion, adhesion and metastasis[32], [46]. We indicated that PGN-SA challenged cancer cells produce more amounts of these cytokines contributing MDA-MB-231 cells to be more invasive and adhesive.

Among those cytokines we analyzed, TLR2-NF-κB activation specially promoted IL-6 and TGF-β higher secretions in invasive MDA-MB-231 cells rather than that in MCF-7 cells. We conclude that IL-6 and TGF-β signal in TLR2-NF-κB-mediated mechanism in MDA-MB-231 cells is very important. Here we further found that PGN-SA effectively triggered STAT3 and Smad3 activation with which respectively were coupled by IL-6 and TGF-β signal. NF-κB, STAT3 and Smad3 activities after PGN-SA stimulation were not on the same time point. Phosphorylation of NF-κB was firstly promoted up to maximum level on 30 min and then phosphorylation of STAT3 and Smad3 were sequentially induced to near maximum level at 4 h and 6 h. These finding indicated that PGN-SA-stimulated TLR2 activation increased NF-κB, STAT3 and Smad3 activities depending on a time pattern. Moreover, TLR2-triggered STAT3 and Smad3 activities could be suppressed by NF-κB inhibition which also decreased IL-6 and TGF-β increments after TLR2 stimulation. Combining these findings, we concluded that TLR2-NF-κB signaling in MDA-MB-231 cells enhances cytokine production involving IL-6 and TGF-β leading STAT3 and Smad3 to sequential activation, respectively. The supernatant of PGN-SA+SN50 treated cells was replaced by the cell culture supernatant with PGN-SA, which could reverse the regression of STAT3 and Smad3 activities previously inhibited by SN50. Thus, STAT3 and Smad3 sequential activities in the cancer cells depended on IL-6 and TGF-β secreted in the supernatant and required NF-κB activity. Collectively, we illustrated a TLR2-mediated mechanism in metastatic breast cancer cell challenged by PGN, a major surface component of Gram-positive bacteria. TGF-β-Smad3 signaling acts as critical role in breast cancer cell invasion and EMT[34]. IL-6 triggers malignant features in human ductal breast carcinoma and IL-6-STAT3 signaling cascade is important for epithelial tumorigenesis and dissemination[33], [43]. Our findings indicate that PGN-stimulated TLR2 activation in highly metastatic breast cancer MDA-MB-231 cells induced extensive molecular activations involving three important transcriptional factor activities which are essential for control of cancer cell malignant properties[34], [47], [48].

Taken together, high level TLR2 expressed in metastatic human breast cancer cells and mediated a bacterial component, PGN, to stimulate NF-κB activity resulting in STAT3 and Smad3 sequential activation, which contributed to invasiveness and adhesiveness. Our study reveals a direct relationship of cancer cells with pathogenic bacteria, suggesting a TLR2-mediated mechanism in bacterial infection-associated human breast cancer metastasis. Since many cancer patients with weaken immune systems appear to be susceptible to bacterial infection and surgical contamination and there are always sporadic serious or light cases of surgical infection in clinic every year[3], [5], [49], our findings pointed importance of antibiotic therapy to treat breast tumor with bacterial infection especially of *Staphylococcus aureus,* a notable drug-resistant pathogen that easily occur in clinic. In addition, as some bacterial vaccine and adjuvant used in cancer treatment usually contain TLR2 agonist, this study is also relevant to genetic strategy for bacterial vaccine and adjuvant design in cancer immunotherapy.
